# Memory and exploratory behavior impairment in ovariectomized Wistar rats

**DOI:** 10.1186/s12993-018-0146-7

**Published:** 2018-07-16

**Authors:** Sefirin Djiogue, Armando Blondel Djiyou Djeuda, Paul Faustin Seke Etet, Germain Jean Magloire Ketcha Wanda, Rudig Nikanor Djikem Tadah, Dieudonne Njamen

**Affiliations:** 1 Laboratory of Animal Physiology, Department of Animal Biology and Physiology, Faculty of Science, University of Yaounde I, P.O. Box 812, Yaoundé, Cameroon; 2 Department of Psychology, Faculty of Arts, Letters and Social Science, University of Yaounde I, P.O. Box 7011, Yaoundé, Cameroon; 3 Center for Sustainable Health and Development, University of Ngaoundere, P.O. Box 454, Ngaoundere, Cameroon

**Keywords:** Ovariectomy, Memory, Object recognition, Spatial memory, Ovarian hormones, Wistar rats

## Abstract

**Background:**

Estrogen deficiency is linked to changes in several physiological processes, but the extent to which it associates with cognitive changes in menopause context is controversial.

**Rationale:**

We evaluated the impact of ovariectomy on memory processes and normal exploratory behavior in Wistar rats.

**Methods:**

Young adult rats (4–6 months) were either ovariectomized (OVX group) (N = 10), sham operated (N = 10), or untouched (naïve controls) (N = 8). Afterwards, they were monitored for 12 weeks during which their cognitive functions were evaluated at first week (S1), second (S2), every 3 weeks (S5, S8) and then at week 12 (S12) using: (i) object recognition test to evaluate the short-term and long-term non-spatial memory; (ii) the object placement test to assess the spatial memory; and (iii) normal exploratory behavior components like locomotor and vertical activities in an open field arena.

**Results:**

Marked changes in ovariectomized rats were observed in long-term non-spatial memory (~ 40% change vs. naïve and sham, P < 0.001) and spatial memory (~ 30% change, P < 0.05) from S2. Instead, from S5 the exploratory behavior was affected, with decreases in line crossing and rearing episode numbers (~ 40% change, P < 0.01), and in the time spent in the center of open field arena (~ 60% change, P < 0.01).

**Conclusions:**

Our findings support the involvement of sex hormones in cognitive functions in female rats and suggest that controversy on the importance of cognitive affections in menopause context may emerge from differences between short-term and long-term memory processes.

## Background

Menopause is a normal component of aging in women accompanied by clinical signs and higher risk for developing diseases such as osteoporosis, cardiovascular diseases, solid cancers, and neurodegenerative diseases [1]. Menopause and the clinical concerns it raises emerge from definitive cessation of menstrual cycle resulting from the cessation of ovarian functions, including the production of estrogens and progesterone. The cessation of ovarian functions affects most physiological processes, including cognitive and motor functions, although, the extent to which central nervous system functions are affected in postmenopausal women is not clear. However, it is widely accepted that compromised memory and motor functions reported in menopausal women may emerge mainly from declines in steroid hormones’ levels, particularly estradiol [2]. Notably, brain structures like the hippocampus, a key player in learning and memory processes, need high levels of estrogens obtained partly by intrathecal production [2, 3].

In the last decades, experimental models of menopause have been used for getting translationally relevant insights in the role of estrogens in cognitive and movement disorders [4, 5]. Ovariectomy is widely used to induce a menopause-like status in laboratory rodents. It results in definitive cessation of ovarian hormones’ production [6], and mimics some features of human menopause. There is controversy on the specific features with translational potential in this model, which contributes to disagreements on the importance of cognitive affections in menopause context [7]. Overall, research reports in rodents and non-human primates suggest that sexual hormones are key players in the maintenance of cognitive abilities [5, 8]. Studies in ovariectomized animals showed significant alterations in the structure and function of hippocampal and cortical circuits accompanied by poor performance in several cognitive tasks [9–12]. These observations were confirmed in humans, as estrogen therapy improved performances in cognitive tasks, verbal memory, and executive functions of perimenopausal women [13]. Many reports confirmed the beneficial effects of estrogen replacement therapy on various physiological functions in ovariectomized animals as well [10, 13]. However, controversy remains on the extent of alterations mediated by ovariectomy in these physiological functions. For instance, although various studies suggest that ovariectomy may impair object recognition ability [10, 14], the importance of such affection is controversial. It was hypothesized that such controversy may emergence from differences in experimental protocols, particularly in time intervals between ovariectomy and object recognition testing [9, 15].

Surprisingly, considering the importance and translational potential of information on the effects of chronic ovarian hormone deprivation on memory processes and other cognitive functions, data comparing the effects of ovariectomy on short-term and long-term memory in rodents, are scarce. Research in rodents and non-human primates’ shows that gonadals hormones are beneficial for the maintenance of cognitive abilities [5, 8]. However, it should be noted that most animal research are based on the replacement of gonadal hormones, usually estrogens, with animals ovariectomized (OVX). Few studies have directly investigated the effects of OVX on short-term or long-term memory [11, 14] or locomotor and exploratory activity in rodents. One study conducted throughout 7 weeks have assessed the short-term memory tasks (OR and OP) of ovariectomized young and intact rats for 7 consecutive weeks. In this study, we wanted to describe the evolution of both short-term and long-term memory over a longer study period. On this basis, we assessed the effects of ovariectomy on normal exploratory behavior, as well as changes in short-term and long-term memory in Wistar rats for 12 weeks.

## Methods

### Animals

Female Wistar rats (N = 28, 4–6 months old, 190–220 g) were obtained from the animal facility of the University of Yaoundé I (Cameroon). Rats were group housed (N = 4 or 5) in standard cages under 12:12 h light–dark cycle, with ad libitum access to food and tap water. Rats were acclimatized to the testing room environment (for 1 week), then they were randomly divided into three groups: (i) ovariectomized animals (N = 10) (OVX group); (ii) sham-operated animals (N = 10); and naïve control animals (N = 8).

All procedures were approved by institutional review board. Animals were handled in accordance with European Commission’s Guidelines for Laboratory Animal Use and Care (EEC Directive of 1986; 86/609/EEC).

### Ovariectomy

Surgery was performed under anesthesia induced by Valium (10 mg/kg, i.p) and ketamine (50 mg/kg, i.p), using standard procedures. Briefly, anesthesia was confirmed by reduced respiratory rate and absence of response to gentle pinching of footpad. Ventral incision was made through the skin on the right flank. In the OVX group, the ovary, oviduct, and top of the fallopian tubes were clamped and removed. In sham-operated rats, same surgical procedure was performed, but ovaries were just palpated, not removed. Skins and abdominal walls of animals of both groups were sutured, and animals were returned to their cages.

### Memory and exploratory behavior assessment

#### Procedures

After the recovery period (1 week), open field (OF), object recognition, and object placement tests were performed 1, 2, 5, 8 and 12 weeks after surgery (S1, S2, S5, S8, and S12). Tests were performed during 3 consecutive days: OF testing in day 1 (used also as animal habituation phase to the arena), object recognition test in day 2, and object placement test on day 3. The apparatus consisted of a wooden box (40.5 × 40.5 cm basis, 30-cm walls’ height), with a computerized camera on top (45°, for capturing both vertical and horizontal activities in the arena). The monitor was placed in an adjoining room. Throughout testing, the door of the testing room was closed, and animals were video recorded. The arena was cleaned with 70% ethanol between rats.

#### Memory evaluation

**Fig. 1:**
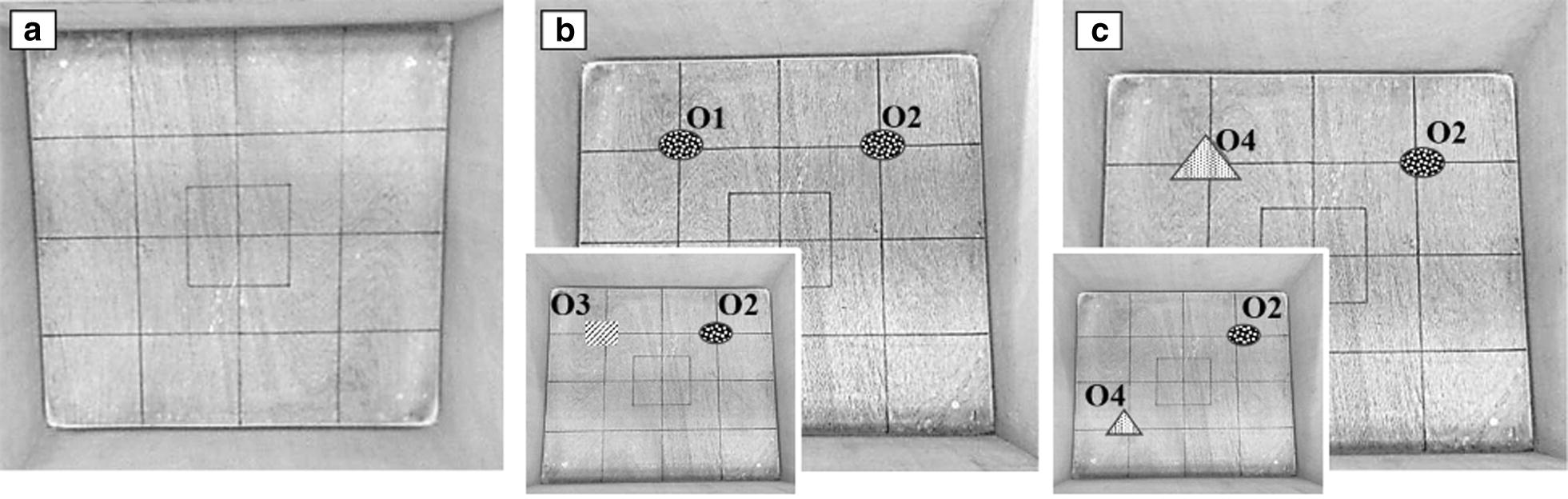
Testing procedure. **a** Habituation phase (day 1). **b** Object recognition assessment (day 2): habituation to objects (**b**) and object recognition test (inset). **c** Spatial memory assessment (day 3): habituation to objects (**c**) and object recognition test (inset)

Testing procedures were based on standard protocols for object recognition and object placement tests, whose tasks are based on the natural affinity of rats for novelty [10, 16, 17]. Four plastic objects (~ 4–6 cm height and width,) were used: two identical objects (O1 and O2) and two objects differing in color and shape (O3 and O4) were used. Memory evaluation was performed in 3 phases: habituation phase, object recognition evaluation phase, and object placement evaluation phase (Fig. 1).

##### Habituation phase (day 1)

Rats explored an empty open field arena (Fig. 1a) similar to OF test procedures [18]. In this phase and in each of the following, a rat was introduced in the open field arena and allowed 5-min to explore the arena and its objects (where applicable). Its exploratory activities were video recorded for 5 min, then scored offline.

##### Object recognition assessment (day 2)

**Fig. 2:**
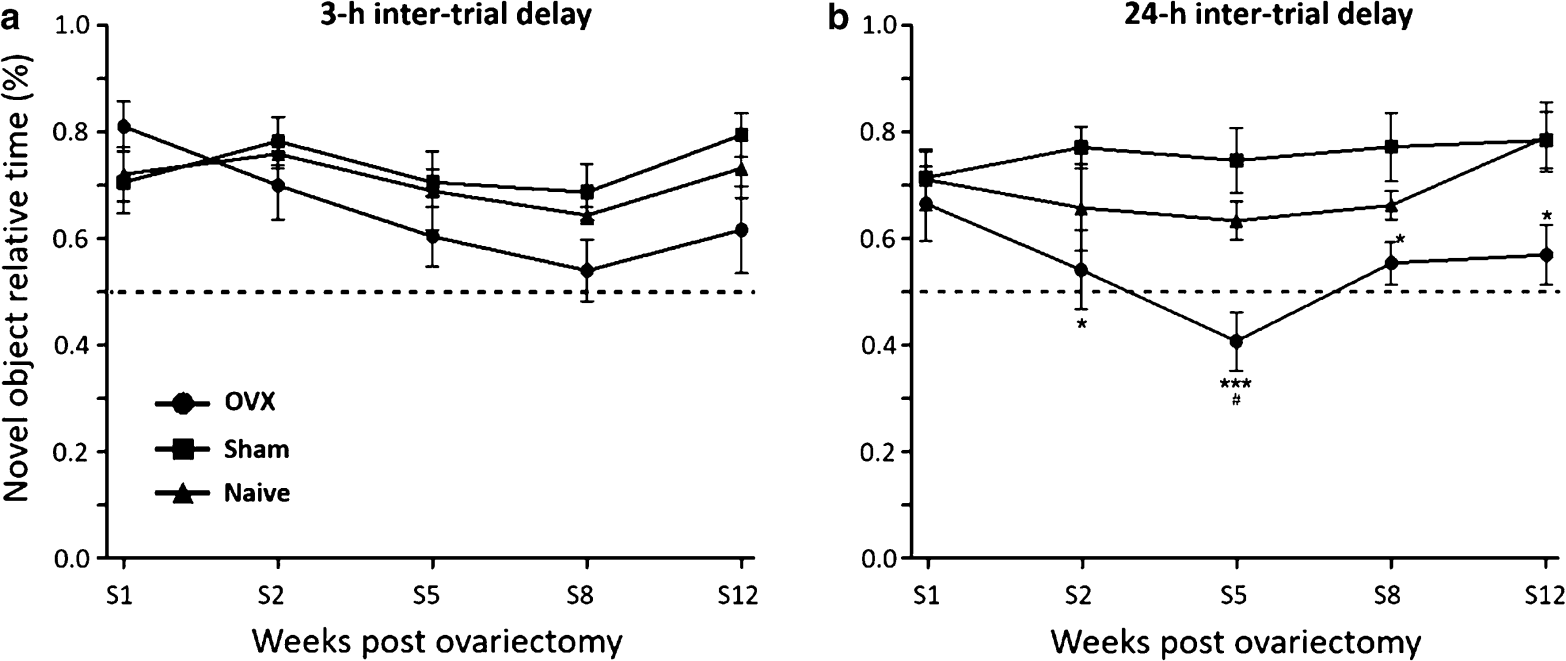
Object recognition assessment. Relative exploration time of novel objects (% of total object exploration time) throughout the first 12 weeks following surgery with 3-h inter-trial time (**a**) or with 24-h inter-trial time (**b**). Treatment groups: ovariectomized animals receiving vehicle solution (OVX) (N = 10), sham operated animals (N = 10), and naïve animals (N = 8). Dashed line indicates chance performance of task, which is the same amount of time spent exploring the old and novel object. ANOVA followed by Bonferroni test: *P < 0.05 vs. sham group; ***P < 0.001 vs. sham group. Data are mean ± SEM

Rats explored the same arena, but with two identical objects (O1, O2) placed as shown in Fig. 2b). After 3-h (short-term memory assessment) or 24-h (long-term memory assessment), the animals explored the arena again, but object O1 was replaced by a novel object (O3) (Fig. 2b inset).

##### Object placement assessment (day 3)

Rats explored the same arena, but object O3 was replaced by a novel object (O4) placed at the same location (Fig. 2c). After 3-h, the animals explored the arena again, but object O4 was placed in a new location (Fig. 2c inset).

##### Data offline scoring

Object exploration was when the subject sniffed, whisked at, or looked at the object from less than 2 cm. Object relative time (exploration ratio) was its exploration time expressed as a percent of total object exploration time.

#### Exploratory behavior evaluation

Habituation phase recordings (OF test) were scored offline. Locomotor activity was evaluated by measuring line crossing, i.e. the number of times the rat crossed grid lines drawn on the flour with all four paws. Other exploratory behavior parameters scored included the total time spent in the central square of the open field arena (Fig. 1a) (an established indicator of anxiety), the number of rearing episodes (where rats stood on their hind legs) (an established indicator of cognition: danger assessment), and the number of grooming episodes (time spent licking or scratching itself while stationary) (whose absence is an established indicator of depression).

### Statistical analysis

Repeated measures ANOVA followed by Bonferroni test was used to evaluate the statistical significance of differences between ovariectomized, sham, and naïve animals’ performances in the various tests. Two factors were considered: the experimental group and the week Differences with P ˂ 0.05 were considered significant. Data were expressed as mean ± SEM.

## Results

### Object recognition

#### Short-term memory

Figure 2a shows the effects of ovariectomy on short-term object recognition ability in rats, throughout the first 12 weeks following surgery. In the first week, all groups spent more time in the exploration of the novel object than in the exploration of the old one. Afterwards, while the sham and naïve animals maintained their novel object-old object time ratios above 0.7, the ratios of OVX group were decreased from 0.8 to 0.6 at week 12. This change was not statistically significant (Fig. 2a). Analysis of the post-surgical ratios with two-way ANOVA (group vs. week) showed a significant group (F = 2.72, P < 0.05) and week (F = 2.73, P < 0.05) effect, without a significant group—week interaction. No statistically significant difference was observed between sham and naive groups.

#### Long-term memory

Figure 2b shows the effects of ovariectomy on long-term object recognition ability in rats, throughout the first 12 weeks following surgery. As observed for short-term memory assessment, all groups spent more time in the exploration of the novel object than in the exploration of the old one in the first week. Afterwards, sham and naïve animals kept novel object-old object time ratios above 0.7 while OVX group ratios were lower than 0.6 (Fig. 2b). At weeks 2 (t = 2.950, P < 0.05), 5 (t = 4.345, P < 0.001), 8 (t = 2.802, P < 0.05), and 12 (t = 2.756, P < 0.05) post-surgery, animals of the OVX group significantly failed to discriminate between the old and new objects. Analysis of the post-surgical ratios by two-way ANOVA showed a significant group effect (F = 16.33, P < 0.0001), without significant week effects or group—week interactions. No statistically significant difference was observed between sham and naive groups.

### Object placement

**Fig. 3:**
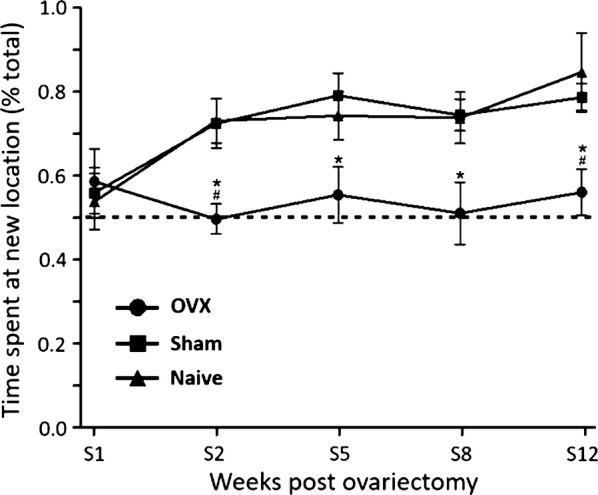
Spatial memory assessment. Relative time spent in the new location of the object relocated (% of total object exploration time) throughout the first 12 weeks following surgery. Treatment groups: ovariectomized animals receiving vehicle solution (OVX) (N = 10), sham operated animals (N = 10), and naïve animals (N = 8). Dashed line indicates chance performance of task, which is the same amount of time spent exploring the old and novel object. ANOVA followed by Bonferroni test: *P < 0.05 vs. sham group; ^#^P < 0.05 vs. naive group. Data are mean ± SEM

Figure 3 shows the effects of ovariectomy on the exploration ratio of the new location, i.e. the ratio of the new location exploration time to old + new location exploration times, throughout the first 12 weeks following surgery in rats. In the first week post-surgery, all animals discriminated the novel location from the old one. From week 2 onward, sham and naïve groups’ exploration ratios increased from 0.55 to more than 0.8 at week 12, while OVX group ratios remained lower than 0.5 (t = 2.971, P < 0.05 vs. the other groups). Post-surgical, two-way ANOVA of the ratios showed significant group (F = 14.40, P < 0.001) and week (F = 3.303, P < 0.05) effects, but no significant group—week interaction. No statistically significant difference was observed between sham and naive groups.

### OF test

#### Line crossing and rearing

**Fig. 4:**
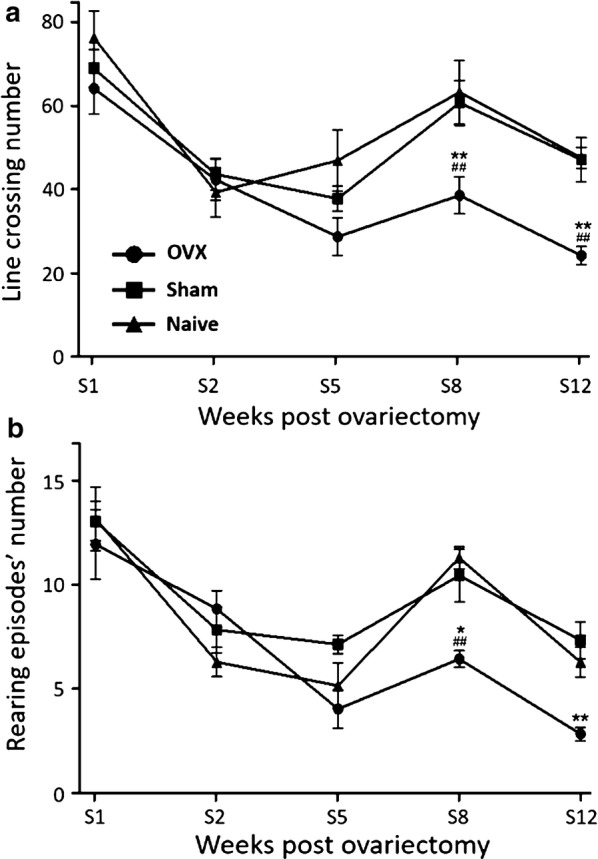
Locomotor and vertical activities. Number of line crossing (**a**) and rearing episodes (**b**) throughout the first 12 weeks following surgery. Treatment groups: ovariectomized animals receiving vehicle solution (OVX) (N = 10), sham operated animals (N = 10), and naïve animals (N = 8). ANOVA followed by Bonferroni test: *P < 0.05, **P < 0.01 vs. sham group; ^##^P < 0.01 vs. naive group. Data are mean ± SEM

Figure 4a shows the effects of ovariectomy on line crossing number in rats, throughout the first 12 weeks following surgery. In the first 2 weeks, the number of line crossing was comparable in all groups. However, from week 5 onward, line crossing number increased markedly in sham and naïve groups (t = 3.373, P < 0.01 vs. baseline values), but not in OVX group (t = 3.214, P < 0.01) (Fig. 4a). Postsurgical analysis using two-way ANOVA showed significant group (F = 15.02, P < 0.0001) and week effects (F = 25.51, P < 0.0001) and no significant group—week interaction. No statistically significant difference was observed between sham and naive groups.

#### Rearing episodes

Figure 4b shows the effects of ovariectomy on rearing episodes’ number in rats, throughout the first 12 weeks following surgery. In the first 7 weeks, changes in rearing episodes’ number were comparable in all groups. However, from week 8 onward, ovariectomized animals displayed significant decreases compared to sham and naïve groups (t = 3.380, P < 0.01) (Fig. 4b). Postsurgical analysis using two-way ANOVA showed significant group (F = 7.481, P < 0.0001) and week effects (F = 28.90, P < 0.0001) and a significant group—week interaction (F = 2.509, P < 0.05). No statistically significant difference was observed between sham and naive groups.

#### Central OF arena time

**Fig. 5:**
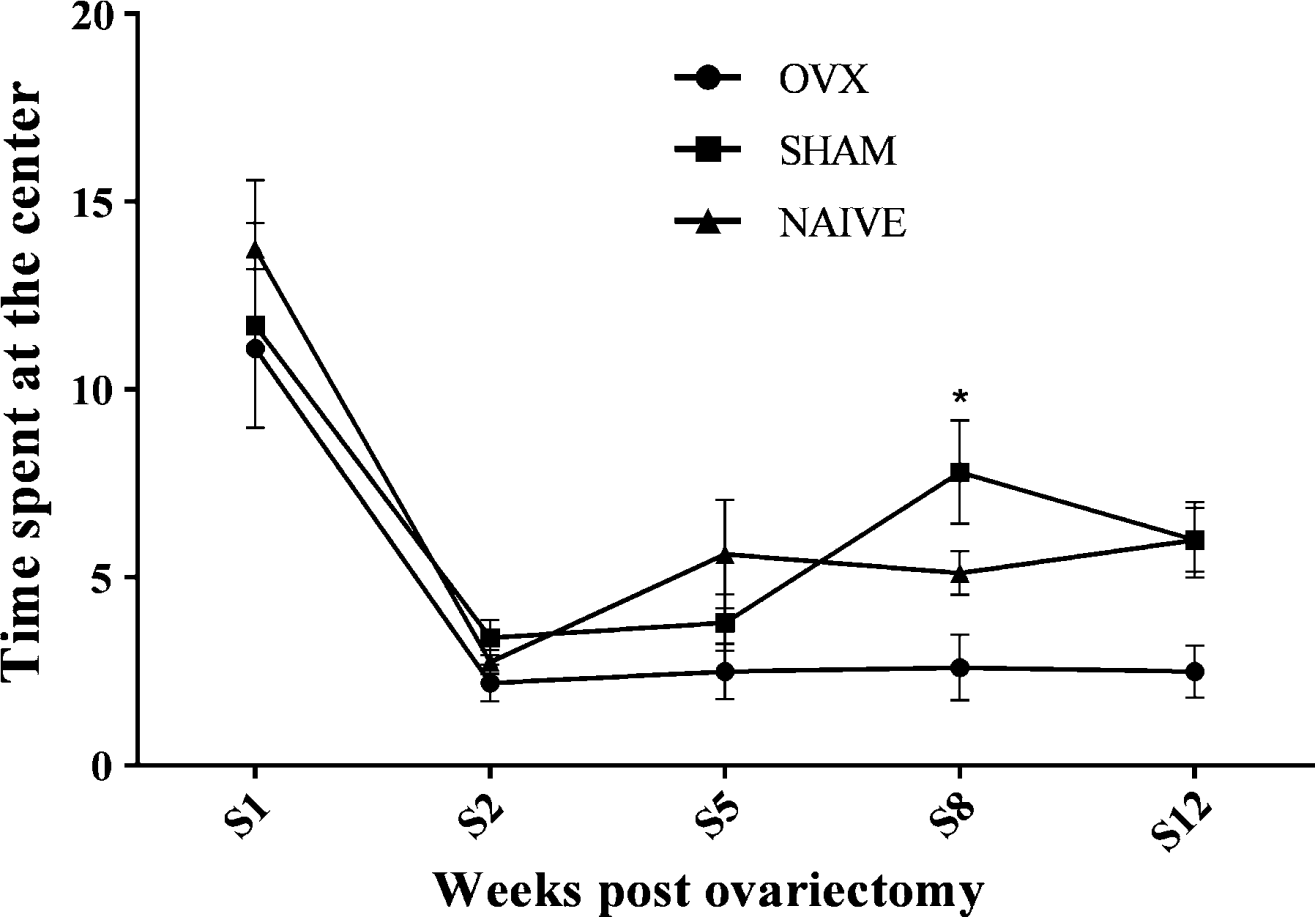
Time in the center of the OF arena. Time spent at the center throughout the first 12 weeks following surgery. Treatment groups: ovariectomized animals receiving vehicle solution (OVX) (N = 10), sham operated animals (N = 10), and naïve animals (N = 8). ANOVA followed by Bonferroni test: *P < 0.05 vs. naive group. Data are mean ± SEM

Figure 5 shows effects of ovariectomy on the time spent at the center of the OF arena in rats, throughout the first 12 weeks following surgery. In the first 7 weeks, changing in the time spent at the center were comparable in all groups. However, from week 8 onward, ovariectomized animals displayed significant decreases compared to sham group (t = 2.940, P < 0.05) (Fig. 5). Postsurgical analysis using two-way ANOVA showed significant group (F = 5.745, P < 0.01) and week effects (F = 24.12, P < 0.001) and no significant group—week interaction. No statistically significant difference was observed between sham and naïve groups.

## Discussion

Our results showed declines of performances in cognitive and motor tasks, and affections of components of normal exploratory behavior in ovariectomized rats. Short-term (3-h inter-trial delay) and long-term (24-h inter-trial delay) non-spatial memory (object recognition) and spatial memory (object placement) started to decline as early as 2 weeks after surgery. Similarly, the numbers of line crossing and rearing episodes were significantly reduced from the 8th week post-surgery. Altogether, these observations suggest that ovarian hormones play a key role in the preservation of cognitive and motor functions in female rats. Our findings corroborate the previous reports suggesting that ovariectomy affects significantly the cognitive processes [10, 18, 19]. The decrease in line crossing (indicator of rodent natural drive to explore a novel environment) and rearing episodes numbers (assessment of the presence of potential environmental dangers) observed in ovariectomized rats in this study also indicate cognitive impairment [20, 21], as well as mood affections [22]. Decreases in the time spent in the center of the open field arena, a well-established indicator of anxiety in rodents [22–25], also suggested an increased anxiety in ovariectomized rats in this study.

The analysis of the relative time spent exploring the novel object in short-term memory evaluation with two-way ANOVA showed significant group and week effects, without significant group-week interactions. This observation suggests that ovariectomy was associated with a decline in performance in object recognition test whose severity increased with time. On the same hand, the evaluation of short-term spatial memory revealed an increasing trend in the time spent exploring the new location in sham and naïve animals, which was lost in ovariectomized animals, where the novel area exploration remained constant. These findings indicate a direct link between estrogen bioavailability and processes driving the plasticity of short-term memory in rats. Instead, the analysis of the relative time spent exploring the novel object in long term memory evaluation with two-way ANOVA showed a significant group effect, but without significant week effect or group—week interactions. This finding also suggests that ovariectomy was associated with a decline in object recognition, but it indicates an indirect link between estrogen depletion and long-term memory affections. Thus, overall, long-term memory affections were more severe but unpredictable in this study, while short-term memory affections were mild for a long time but increased in severity. This is not surprising considering that short-term memory depends more on perirhinal, entorhinal and medial prefrontal cortical areas while long-term memory relies heavily on the hippocampal formation in mammals [26, 27], and given that estrogens’ importance is area-specific in the brain, due to area-specific repartition of subtypes and expressions of estrogen receptors [9, 13, 27–30].

These findings support the hypothesis that controversy on the importance and effects of sustained estrogen depletion on memory and other cognitive processes may emerge from differences in time intervals between ovariectomy and object recognition testing, among other parameters differing between the experimental protocols used in the available reports [10, 16]. Thus, future studies attempting to confirm the available data should consider harmonizing the experimental protocols with protocols used in the studies that produced them.

Our results revealed an alteration of the spatial and nonspatial memory processes, both short and long term, of intact rats, compared to OVX rats observed from week 2–12 post OVX. Knowing that OR and OP are hippocampal-dependent tasks [31], our results suggest that estradiol would act as a regulator of cellular processes in the hippocampus. The mechanisms underlying the rapid effects of estrogen also remain to be elucidated. However, it has been suggested that E2 may produce its effects by binding to its ERα and ERβ intracellular canonical receptors located in the dendritic spines, dendrites, axons and nucleus of the pyramidal neurons of the hippocampus [28]. The classical mechanism by which E2 exert his effects involve the formation of an E2-ER complex in the cytoplasm, its translocation into the nucleus, followed by its attachment to the estrogen response element. (ERE) which then initiates transcription. However, since this mechanism is too slow and insufficient to explain by itself the multiple effects of E2 on the hippocampus, other mechanisms have also been suggested. E2 may also influence neuronal function through the rapid activation of cell signalling cascades such as phosphatidylinositol 3-kinase (PI3 K) and extracellular signal-regulated kinase (ERK) [10], by induction of post-translational epigenetic modifications (histone acetylation and DNA methylation) [30] or by initiating the synthesis of mTOR proteins (mammalian Target Of Rapamycin). Moreover, it is currently accepted that E2 may influence the hippocampus via the interaction between ERs and neurotransmitter receptors (NMDA receptors for example) and/or by attachment to new estrogen receptors located in the plasma membrane and/or synaptic terminals (GPR30) [32]. It has been proposed that perirhinal, entorhinal and medial prefrontal cortical areas are crucial for object memory, whereas spatial recognition involves hippocampal pathways in rats [27]. Thus, this suggests that, effects of reduction of the ability to recognize the object in OVX rats in our study are not limited to, or specific to, hippocampus pathways, but rather extend to several other brain structures.

Few studies have investigated the mechanisms by which E2 promotes the exploratory behaviour of rats. The hypothesis currently accepted is that E2 would act mainly via ERα, ERβ being little or not at all involved [33, 34]. However, the mechanisms by which ERα influences locomotor activity are unclear. ERα may still act by modulating the activity of many neurotransmitters, including dopamine and/or serotonin [35]. Indeed, estrogens are able to regulate different stages of dopaminergic functioning, including the release of dopamine, its metabolism and the functioning of its pre- and postsynaptic proteins [36, 37]. In addition, E2 may also acts on the dopaminergic system via non-genomic mechanisms of action, not involving ERα or ERβ [38].

## Conclusions

Our data suggest that memory and locomotor activity may be impaired by ovariectomy in female rats, indicating pivotal roles for ovarian hormones in memory processes and motor functions in rodents. The mechanisms by which these effects are mediated are not clear. Thus, further studies are required to substantiate these findings, considering the translational potential and implications. For instance, it would be interesting to characterize the receptors, the downstream cascades of signaling molecules, and other potential pharmacological targets involved.
